# Lugdunin production and activity in *Staphylococcus lugdunensis* isolates are associated with its genotypes

**DOI:** 10.1128/spectrum.01298-23

**Published:** 2023-09-21

**Authors:** Shih-Cheng Chang, Cheng-Yen Kao, Lee-Chung Lin, Jazon Harl Hidrosollo, Jang-Jih Lu

**Affiliations:** 1 Department of Laboratory Medicine, Linkou Chang Gung Memorial Hospital, Taoyuan, Taiwan; 2 Department of Medical Biotechnology and Laboratory Science, College of Medicine, Chang Gung University, Taoyuan, Taiwan; 3 Institute of Microbiology and Immunology, College of Life Sciences, National Yang Ming Chiao Tung University, Taipei, Taiwan; 4 Department of Medicine, College of Medicine, Chang Gung University, Taoyuan, Taiwan; Michigan State University, East Lansing, Michigan, USA

**Keywords:** *Staphylococcus lugdunensis*, lugdunin, genotype, *S. aureus*

## Abstract

Lugdunin produced by *Staphylococcus lugdunensis* has been shown to have broad inhibitory activity against Gram-positive bacteria; however, lugdunin activity among *S. lugdunensis* isolates and its association with different *agr*, SCC*mec*, and sequence types remain unclear. We used matrix-assisted laser desorption ionization-time-of-flight mass spectrometry to identify *S. lugdunensis* and collected 202 *S*. *lugdunensis* samples for further assays. Agar spot tests were performed to characterize *S. lugdunensis* lugdunin production and activity. Multilocus sequence typing, SCC*mec,* and *agr* genotyping were performed on *S. lugdunensis*. In all, 91 S*taphylococcus aureus* strains with varying vancomycin susceptibilities were used to examine lugdunin activity in *S. lugdunensis*. In total, 48 *S*. *lugdunensis* strains (23.8%) were found to be oxacillin-resistant *S. lugdunensis* (ORSL), whereas 154 (76.2%) were classified as oxacillin-sensitive *S. lugdunensis* (OSSL). Moreover, 16 (33.3%) ORSL and 35 (22.7%) OSSL strains showed antibacterial activity against *S. aureus*. Our data showed that most lugdunin-producing ORSL strains (14/48, 29.2%) were of ST3-SCC*mec* V-*agr* II genotypes, whereas most lugdunin-producing OSSL strains (15/154, 9.7%) were of ST3-*agr* II, followed by ST1-*agr* I (10/154, 6.5%). Our data also revealed that lugdunin exhibited weak inhibitory activity against the VISA ST239 isolate. In addition, we observed that ST239 VSSA was more resistant to lugdunin than ST5, ST59, and ST45 VSSA. Taken together, our data pioneered the epidemiology of lugdunin production in *S. lugdunensis* isolates and revealed its association with genotypes. However, further molecular and bioinformatics investigations are needed to elucidate the regulatory mechanisms of lugdunin production and activity.

**IMPORTANCE:**

Lugdunin is active against both methicillin-resistant *Staphylococcus aureus* and vancomycin-resistant enterococci by dissipating their membrane potential. However, the association of lugdunin activity with the genotypes of *Staphylococcus lugdunensis* has not been addressed. Here, we show the high prevalence of lugdunin-producing strains among ST1 (83.3%), ST2 (66.7%), and ST3 (53.3%) *S. lugdunensis*. Moreover, we identified the antibacterial activity of lugdunin-producing strains against VISA and hVISA. These results shed light on the potential application of lugdunin for the treatment of drug-resistant pathogens.

## INTRODUCTION

Infections caused by highly antibiotic-resistant bacteria continue to pose a threat to public health because they represent one of the leading causes of morbidity and mortality worldwide, including in developed countries (1). Antimicrobial resistance (AMR) makes treatment strategies more difficult because clinically available antibiotics are no longer effective. Unfortunately, the AMR burden is coupled with increasing costs and pressure on pricing for drug development (2). Therefore, diminishing the supply of new antibiotics that can replace these ineffective drugs remains an ongoing problem. Therefore, urgent efforts and immediate development to discover new antibiotics from potentially underexplored niches are required.

Natural products play promising roles in the development of therapeutics against infectious diseases (3). Recently, the human microbiome has been recognized as a niche from which bioactive secondary metabolites can be isolated (3). *Staphylococcus aureus* is a disease-causing opportunistic pathogen that resides in the nasal cavities of 30% of the population and is prone to multidrug resistance (4, 5). To combat *S. aureus* infections, a notable bacterium being studied for its potential probiotic activities is a coagulase-negative *Staphylococcus*, *Staphylococcus lugdunensis* (6, 7). In recent years, clinical reports have shown that it can also be an emerging pathogen because it causes life-threatening invasive infections, such as endocarditis and periprosthetic joint infections (8). However, little attention has been paid to *S. lugdunensis* as a potential probiotic (6). In 2016, using functional and chemistry-based approaches, Zipperer et al. discovered that *S. lugdunensis* produced a novel antibiotic called lugdunin, which exhibited broad bioactivity against Gram-positive bacteria, including methicillin-resistant *S. aureus*, vancomycin, and glycopeptide-intermediate-resistant *S. aureus* strains (3, 9). *S. lugdunensis* IVK28 has a particularly strong capacity to prevent the growth of *S. aureus* (9).

Lugdunin is a thiazolidine-containing cyclic peptide antibiotic that is synthesized non-ribosomally and belongs to a new class of antibiotics (9). The analysis of a transposon insertion site in the mutant M1 strain revealed the disruption of an uncharacterized gene encoding a putative non-ribosomal peptide synthetase (NRPS) (9). The operon responsible for lugdunin biosynthesis consists of four NRPS genes (*lugA*, *B*, *C*, and *D*) encoding adenylation domains for five amino acids and is exclusively found in *S. lugdunensis* (9).

Multilocus sequence typing (MLST) is the most widely used genotyping tool in epidemiological studies of *S. lugdunensis* to monitor its prevalence in hospitals (10 – 12). In addition, our previous study showed an association between ST and CRISPR-Cas types in *S. lugdunensis* (13). Among 130 CRISPR-Cas-positive *S. lugdunensis* isolates, type IIIA and type IIC CRISPR-Cas were found in 84 (64.6%) and 46 (35.4%) isolates, respectively (13). Isolates ST1, ST6, ST12, and ST15 harbored type IIIA CRISPR-Cas, whereas isolates ST4, ST27, and ST29 harbored type IIC CRISPR-Cas. None of the 60 ST3 isolates harbored type IIIA or type IIC CRISPR-Cas (13). Currently, clinical attention has been paid to *S. lugdunensis* because of its ability to resist β-lactam antibiotics such as oxacillin (oxacillin-resistant *S. lugdunensis*, ORSL) (11). Thus, SCC*mec* typing is a “gold standard method” to determine resistance to β-lactam antibiotics (14, 15). Most virulence factors in *S. aureus* are closely regulated by the *agr* locus, which encodes a two-component signaling pathway that can be divided into four major *agr* groups: types I–IV (16). The *agr* locus in *S. lugdunensis* was recently shown to play a role in biofilm formation and resistance to host immune killing (17, 18). Hence, despite well-reported evidence regarding the chemical structure and biosynthetic gene clusters of lugdunin, the epidemiology of lugdunin-producing strains and the association of lugdunin production with STs, SCC*mec*, and *agr* genotypes are still unknown. Thus, in the present study, we aimed to characterize lugdunin activity in *S. lugdunensis* clinical isolates collected from our hospital and investigate its association with different genotypes.

## MATERIALS AND METHODS

### Bacterial strains

A total of 202 *S*. *lugdunensis* isolates were collected between 2003 and 2014 at Chang Gung Memorial Hospital (Linkou), Taiwan. The sample sources and antimicrobial susceptibility results of 202 *S*. *lugdunensis* isolates are shown in Table S1. All *S. lugdunensis* isolates were identified using a Bruker Biotyper (database 2.0) matrix-assisted laser desorption ionization-time-of-flight mass spectrometry system, according to the manufacturer’s instructions, and *S. lugdunensis* isolates were stored in a tryptic soy broth with 20% glycerol at −80°C until further experiments. Vancomycin susceptibility was determined using the modified population analysis profile/area under the curve (19). In all, 91 *S. aureus* strains containing 28 ST5, 8 ST45, 27 ST59, and 28 ST239, with different vancomycin susceptibilities, were randomly selected and used as test pathogens to examine lugdunin activity in *S. lugdunensis* (20).

### Screening of lugdunin activity by an agar spot test

Basic medium (BM) agar was prepared (1% soy peptone, 0.5% Kat yeast extract, 0.5% NaCl, 0.1% glucose, and 0.1% K_2_HPO4; pH 7.2) as previously described (21). A single colony of *S. aureus* HG001 was inoculated into a 5-mL BM medium tube (17 × 100 mm) and incubated at 37°C with shaking for 18–24 h. The BM agar was then melted and allowed to cool in a 50°C water bath for 0.5–1 h. The BM agar was then supplemented with 2,2′-dipyridyl (2-DP, 200 µM), an iron chelator, and the optical density (OD)_600_ of *S. aureus* was adjusted to 0.125 and added together in the BM agar. The mixture was poured into a Petri dish and allowed to solidify. An *S. lugdunensis* overnight BM medium was prepared, and the *S. lugdunensis* culture was washed twice with 0.1× volume of phosphate-buffered saline and centrifuged at 4,000 rpm for 15 min at room temperature. The supernatant was discarded. The *S. lugdunensis* pellets were adjusted to OD_600_ = 20 using the BM medium, and 10 µL was dropped at the center of the BM agar and allowed to dry. The BM agar was incubated for 48 h, and zone clearing or inhibition was observed. Clear zone inhibition was calculated as the inhibition zone diameter—colony diameter (cm), and the absence of a clear zone of inhibition was considered negative. Strains SL131 and SL118, with known genome sequences, were used as lugdunin-active positive and negative controls, respectively (22, 23). Strain SL85, with strong lugdunin activity (zone size >0.2 cm), was used to examine anti-*S*. *aureus* activity.

### SCC*mec*, STs, and *agr* genotyping

All protocols for SCC*mec*, STs, and *agr* genotyping were adopted from previous studies. For SCC*mec* typing, all ORSL isolates were performed using multiplex PCR (M-PCR 1 and M-PCR 2) and were interpreted as described previously (24). Briefly, the 10 µL reaction mixtures of M-PCR 1 contained 10 ng chromosomal DNA, M1 oligonucleotide primers (0.1 M), and Phusion Flash High-Fidelity PCR Master Mix (Thermo Fisher Scientific, USA) at a final volume of 10 µL. A three-step thermal cycling protocol was used with an initial denaturation step (94°C, 2 min), followed by 30 cycles of denaturation (94°C, 1 min), an annealing step (50°C, 1 min), and an extension step (72°C, 1 min); and a final extension step at 72°C for 10 min. The 10 µL reaction mixtures of M-PCR 2 were the same as those for M-PCR 1 except that the annealing temperature was raised to 60°C for 1 min (24). For MLST typing of *S. lugdunensis*, seven housekeeping genes (*aroE*, *dat*, *ddl*, *gmk*, *ldh*, *recA*, and *yqiL*) were used, and the fragments were amplified using the primers as described previously (10). The selection of these housekeeping genes relied on their use in MLST schemes of *S. aureus* (25). The Pasteur MLST sequence definition database contains allele and profile data of *S. lugdunensis* (https://bigsdb.pasteur.fr/staphlugdunensis/). The ST of each *S. lugdunensis* isolate was determined using the search tool with a combination of *S. lugdunensis* loci (26). Two PCR primers (SL_*agr*-F and SL_*agr*-R) were designed to detect the *agr* genes of *S. lugdunensis* isolates (27). In addition, two forward primers and one reverse primer (SL_agr-1-F, SL_agr-2-F/SL_agr-R) were used to determine the *agr* type (27).

### Statistical analysis

We used chi-square tests to compare categorical variables. All statistical analyses were conducted using SPSS version 22.0 (IBM, Armonk, NY, USA). A *P*-value of <0.05 was considered to indicate a statistically significant difference.

## RESULTS

### Association between SCC*mec*, STs, and *agr* genotypes to lugdunin activity

**Fig 1:**
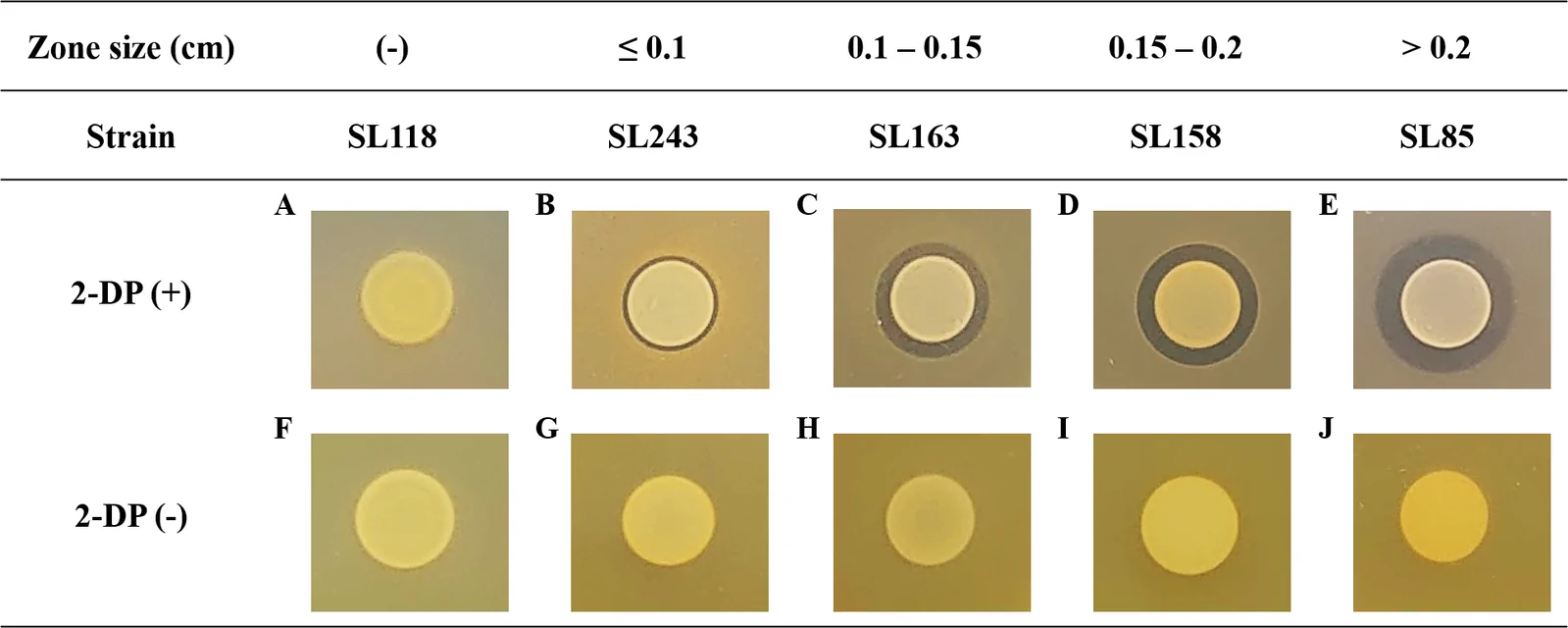
Representative results of lugdunin activity using agar spot test. *S. aureus* HG001 was used to test the anti-*S*. *aureus* activity of lugdunin. SL243 [ST24-*agr* I oxacillin-sensitive *S. lugdunensis* (OSSL)], SL163 (ST2-*agr* II OSSL), SL158 (ST6-*agr* I OSSL), and SL85 [ST3-SCC*mec* V-*agr* II oxacillin-resistant *S. lugdunensis* (ORSL)] were used to represent *S. lugdunensis* with different anti-*S*. *aureus* activity. SL118 (ST6-SCC*mec* II-*agr* I ORSL) was considered a lugdunin-negative control strain. 2,2′-dipyridyl (2-DP), a ferrous iron chelator, was added to induce lugdunin production.

In total, 202 *S*. *lugdunensis* isolates were screened for SCC*mec*, STs, *agr* genotypes, and lugdunin activity. In all, 48 strains were found to be ORSL, whereas 154 were classified as oxacillin-sensitive *S. lugdunensis* (OSSL). A representative result of lugdunin activity is shown in Fig. 1. SL118 without the *lug* operon was used as a lugdunin-negative control strain. Higher lugdunin production by *S. lugdunensis* has been observed under iron-limited culture conditions (9); however, we observed that without treatment with a ferrous iron chelator (2-DP), none of the test strains showed lugdunin activity. However, after 2-DP treatment, SL85 exhibited the highest anti-*S*. *aureus* activity (zone size >0.2 cm), followed by SL158 (0.15–0.2 cm), SL163 (0.1–0.15 cm), and SL243 (≤0.1 cm) (Fig. 1).

Of the 48 ORSL strains, 16 (33.3%) showed a clear inhibition zone against *S. aureus* HG001 and were classified as lugdunin-producing strains (Table 1). Of the 154 OSSL strains, 35 (22.7 %) were classified as lugdunin producers (Table 1). Among the 16 lugdunin-producing ORSL *S. lugdunensis* strains, SCC*mec* type V, VT, and untypable strains were found in 14, 1, and 1 strains, respectively (Table 1). More than 50% (14/27) of the SCC*mec* type V ORSL *S. lugdunensis* strains were lugdunin producers. Nevertheless, no statistical difference in oxacillin susceptibility (*P* = 0.398) and the type of SCC*mec* (*P* = 0.147) was observed between the lugdunin-producing and non-producing strains. Of the 110 *agr* II *S. lugdunensis* strains, 34 (31%) were lugdunin producing; however, only 16 (18%) lugdunin-producing *S. lugdunensis* strains were found in the 89 *agr* I type isolates (*P* = 0.014). ST3 (32 strains) was dominant in the 51 lugdunin-producing *S. lugdunensis* strains, followed by ST1 (10 strains), ST6 (5 strains), ST2 (2 strains), ST24 (1 strain), and ST27 (1 strain) (*P* < 0.001) (Table 1). The majority of the ORSL showed a clear zone measurement of 0.15–0.2 cm (10 strains), followed by >0.2 cm (4 strains) and 0.1–0.15 cm (2 strains) (Table 1). In addition, a clear zone measurement of 0.15–0.2 cm was also observed in the majority of the OSSL strains (18 strains), followed by >0.2 cm (11 strains) and 0.1–0.15 cm (5 strains), whereas only one strain was observed to have a clear zone measurement of ≤0.1 cm (Table 2).

**TABLE 1:** Distribution of lugdunin activity in *S. lugdunensis* with different oxacillin susceptibility, SCC*mec*, *agr*, and MLST types

Oxacillin susceptibility and genotype	Total strain number	Inhibition zone size (cm) of lugdunin-producing strains, no. (%)				Lugdunin non-producing strain number	*P* value
		≤0.1	0.1–0.15	0.15–0.2	>0.2		
OSSL^*c*^	154	1 (0.6)	5 (3.2)	18 (11.7)	11 (7.1)	119 (77.3)	0.398
ORSL^*b*^	48	–^*d*^	2 (4.2)	10 (20.8)	4 (8.3)	32 (66.7)	
SCC*mec* type							0.147
II	16	–	–	–	–	16 (100)	
IV	1	–	–	–	–	1 (100)	
V	27	–	2 (7.4)	8 (29.6)	4 (14.8)	13 (48.1)	
Vt	2	–	–	1 (50)	–	1 (50)	
Untyped	2	–	–	1 (50)	–	1 (50)	
*agr* genotype							0.014
I	89	1 (1.1)	–	9 (10.1)	6 (6.7)	73 (82)	
II	110	–	6 (5.5)	19 (17.3)	9 (8.2)	76 (69.1)	
Other	3	–	1 (33.3)	–	–	2 (66.7)	
MLST^*a*^							<0.001
ST1	12	–	–	7 (58.3)	3 (25)	2 (16.7)	
ST2	3	–	1 (33.3)	1 (33.3)		1 (33.3)	
ST3	60	–	6 (10)	17 (28.3)	9 (15)	28 (46.7)	
ST4	14	–	–	–	–	14 (100)	
ST6	69	–	–	2 (2.9)	3 (4.3)	64 (92.8)	
ST9	4	–	–	–	–	4 (100)	
ST12	4	–	–	–	–	4 (100)	
ST15	3	–	–	–	–	3 (100)	
ST24	1	1 (100)	–	–	–	–	
ST27	26	–	–	1 (3.8)	–	25 (96.2)	
ST29	6	–	–	–	–	6 (100)	

^*a*^ MLST, multilocus sequence typing.

^*b*^ ORSL, oxacillin-sensitive *S. lugdunensis*.

^*c*^ OSSL, oxacillin-resistant *S. lugdunensis*.

^*d*^ The dash (–) denotes the lack of measurable inhibition zones in clinical isolates.

**TABLE 2:** Summary of inhibition zone size and molecular types

Oxacillin susceptibility and genotype	Total strain number	Inhibition zone size (cm) of lugdunin-producing strains, no. (%)				Lugdunin non-producing strain number	*P* value
		≤0.1	0.1–0.15	0.15–0.2	>0.2		
ORSL (*n* = 48)^*a*^	48	–^*c*^	2 (4.2)	10 (20.8)	4 (8.3)	32 (66.7)	0.350
ST3-SCC*mec* V-*agr* II	27	–	2 (7.4)	8 (29.6)	4 (14.8)	13 (48.1)	
ST3-SCC*mec* IV-*agr* II	1	–	–	–	–	1 (100)	
ST3-SCC*mec* VT-*agr* II	2	–	–	1 (50)	–	1 (50)	
ST6-SCC*mec* II-*agr* I	16	–	–	–	–	16 (100)	
Other	2	–	–	1 (50)	–	1 (50)	
OSSL (*n* = 154)^*b*^	154	1 (0.6)	5 (3.2)	18 (11.7)	11 (7.1)	119 (77.3)	<0.001
ST1- *agr* I	12	–	–	7 (58.3)	3 (25)	2 (16.7)	
ST2-*agr* II	3	–	1 (33.3)	1 (33.3)	–	1 (33.3)	
ST3-*agr* II	28	–	3 (10.7)	7 (25)	5 (17.9)	13(46.4)	
ST4-*agr* II	14	–	–	–	–	14 (100)	
ST6-*agr* I	52	–	–	2 (3.8)	3 (5.8)	47 (90.4)	
ST9-*agr* II	4	–	–	–	–	4 (100)	
ST12-*agr* I	4	–	–	–	–	4 (100)	
ST15-*agr* I	3	–	–	–	–	3 (100)	
ST24-*agr* I	1	1 (100)	–	–	–	–	
ST27-*agr* II	24	–	–	1 (4.2)	–	23 (95.8)	
ST29-*agr* II	6	–	–	–	–	6 (100)	
Other	3	–	1 (33.3)	–	–	2 (66.7)	

^*a*^ ORSL, oxacillin-sensitive *S. lugdunensis*.

^*b*^ OSSL, oxacillin-resistant *S. lugdunensis*

^*c*^ The dash (-) denotes the lack of measurable inhibition zones in clinical isolates.

A summary of the number and percentage of the lugdunin-producing strains with their corresponding zone measurements and non-producing strains, when grouped into the STs, SCC*mec*, and *agr* genotypes, is presented in Table 2. Although no statistical difference was observed between the lugdunin production and genotypes of ORSL strains (*P* = 0.350), our data showed that 27 (56.3%) ORSL strains were ST3-SCC*mec* V-*agr* II genotypes, 14 (51.9%) of which were lugdunin producing and 13 (48.1%) were lugdunin-non-producing (Table 2). We observed that 16 ORSL strains were positive for the ST6-SCC*mec* II-*agr* I genotype, and interestingly, none of the 16 (100%) strains were lugdunin producers (Table 2). Only two ORSL strains belonged to ST3-SCC*mec* VT-*agr* II, and one (50%) was a lugdunin producer (Table 2). Finally, only one (100%) ORSL was classified as ST3-SCC*mec* IV-*agr* II-positive and was noted to be a lugdunin-non-producing strain (Table 2). The difference in the distribution of STs and *agr* genotypes was statistically significant between the lugdunin-producing and non-producing OSSL strains (*P* < 0.001). In all, 52 OSSL strains belonged to ST6-*agr* I, and 47 (90.4%) of them were classified as non-lugdunin-producing strains (Table 2). In addition, 28 OSSL strains belonged to ST3-*agr* II, and 15 (53.6%) of them were lugdunin-producing strains (Table 2). Moreover, 24 OSSL strains belonged to ST27-*agr* II, and only one of them produced lugdunin (Table 2). We also observed that the 31 OSSL strains included 14 ST4-*agr* II, 4 ST9-agr II, 4 ST12-*agr* I, 3 ST15-*agr* I, and 6 ST29-*agr* II strains, all of which did not produce lugdunin (Table 2). In all, 12 OSSL strains were also classified as ST1-*agr* I, and 10 (83.3%) were lugdunin-producing strains (Table 2). Lastly, we observed a low prevalence (<5%) of OSSL strains belonging to ST2-*agr* II, ST24-*agr* I, and others (Table 2).

### Lugdunin antibacterial activity against *S. aureus*

We next investigated the antibacterial activity of lugdunin against 91 different *S. aureus* ST types (28 ST5, 8 ST45, 27 ST59, and 28 ST239) and vancomycin susceptibility [vancomycin-susceptible *S. aureus* (VSSA), vancomycin-intermediate *S. aureus* (VISA), and heteroresistant VISA (hVISA)] (Table 3). ST45 hVISA cells were not collected for the lugdunin activity assay. In addition, only one VISA ST239 strain was identified for this test, and the results showed that lugdunin had weak inhibitory activity against VISA. ST5 (two isolates) and ST239 (two isolates) hVISA isolates showed an inhibition zone ranging from 0.1 to 0.15 cm (Table 3). In contrast, lugdunin showed weak inhibition activity (≤0.1 cm) against one ST59 and nine ST239 hVISA isolates. Interestingly, we observed that ST239 VSSA was more resistant to lugdunin than ST5, ST59, and ST45 VSSA (Table 3).

**TABLE 3:** Antibacterial activity of lugdunin against different sequence types and vancomycin susceptibility of *S. aureus*

	Total	No. (%) of isolates showing different ranges of inhibition zones (cm)			
		≤0.1	0.1–0.15	0.15–0.2	>0.2
ST5					
VISA^*b*^	–^*d*^	–	–	–	–
hVISA^*c*^	2	–	2 (100)	–	–
VSSA^*a*^	26	1 (3.8)	14 (53.8)	10 (38.5)	1 (3.8)
ST45					
VISA	–	–	–	–	–
hVISA	–	–	–	–	–
VSSA	8	2 (25)	2 (25)	3 (37.5)	1 (12.5)
ST59					
VISA	–	–	–	–	–
hVISA	1	1 (100)	–	–	–
VSSA	26	5 (19.2)	17 (65.4)	4 (15.4)	–
ST239					
VISA	1	1 (100)	–	–	–
hVISA	12	9 (75)	2 (16.7)	1 (8.3)	–
VSSA	15	13 (86.7)	2 (13.3)	–	–

^*a*^ VSSA, vancomycin-susceptible *S. aureus*.

^*b*^ VISA, vancomycin-intermediate *S. aureus*.

^*c*^ hVISA, heteroresistant VISA.

^*d*^ The dash (–) denotes the lack of measurable inhibition zones in clinical isolates.

## DISCUSSION

In this study, we determined the prevalence of lugdunin-producing *S. lugdunensis* strains and found that the distribution of lugdunin-producing strains was associated with SCC*mec*, *agr*, and ST genotypes. Our lugdunin screening using the agar spot test showed that not all *S. lugdunensis* strains produced lugdunin under the given conditions, strongly suggesting that lugdunin production may be strain specific and associated with STs or other genotypes that could affect lugdunin production and regulation. We analyzed its association with different genotypes and found that the majority of the ORSL lugdunin-producing strains belonged to ST3 and harbored *the agr* II genotype. This ST type was similar to that of the reference strain IVK28 used by Zipperer et al., which also belongs to ST3. The SCC*mec* typing of ORSL revealed that most strains (27, 56.3%) harbored SCC*mec* type V, strongly suggesting that it was community acquired. However, 16 patients (33.3%) had SCC*mec* type II, indicating nosocomial infections. Owing to the low prevalence of ORSL in lugdunin production, the association between SCC*mec* types and lugdunin production remains unclear. The majority of the OSSL lugdunin-producing strains also belonged to ST3-*agr* II. Regarding the association between lugdunin production and STs, our data strongly suggest that the activity of the lugdunin operon differed among STs. However, the organization of the lugdunin operon and the relative gene expression in lugdunin-non-producing ST3-SCC*mec* V-*agr* II are unclear. Our previous study showed a strong association between the CRISPR/Cas system and STs in *S. lugdunensis* (13). These findings strongly suggest an association between the CRISPR-Cas system and lugdunin activity in *S. lugdunensis,* although the role of the CRISPR-Cas system in *S. lugdunensis* remains unknown.

Similar to what we observed in the lugdunin-producing group, a different prevalence rate of lugdunin non-production was observed among STs. This led us to speculate why some *S. lugdunensis* strains failed to produce lugdunin. There are three possibilities: (i) the expression of the lugdunin operon genes in the lugdunin-non-producing group may be low and may not produce lugdunin to exhibit antibacterial activity. (ii) Single-nucleotide polymorphisms or other mutations may occur in the lugdunin operon, leading to truncated or inactive enzymes for lugdunin synthesis. (iii) Other novel gene clusters, ligand enhancers, or inhibitors may also directly or indirectly regulate lugdunin production and activity. Thus, further investigation is required.

The quorum-sensing *agr* system is a major element in pathogenicity regulation and biofilm formation in *S. aureus* (28). *S. lugdunensis* shares genomic features with *S. aureus* (29), and our results showed that the *agr* system might play a role in lugdunin production. To date, the *agr* locus in *S. lugdunensis* has been shown to play a role in biofilm formation and resistance to host immune killing; however, its role in lugdunin production has not been investigated (17, 18). Therefore, our study provides a potential role of *agr* in the regulation and production of *S. lugdunensis* lugdunin. However, the regulatory mechanisms for lugdunin production by *agr* remain to be investigated.

It is also worth noting that lugdunin produced by SL85 was active against various sequence types of *S. aureus* with different vancomycin susceptibilities. Our results showed that lugdunin had relatively weak antibacterial activity against ST239 VISA compared with that against hVISA and VSSA. However, it is promising baseline data to prompt the use of lugdunin in clinical practice. The synergistic effect of lugdunin antibacterial activity combined with other antibiotics is worth investigating in the future. Hort et al. reported that the alteration of the cell wall architecture with a thick cell wall and low cross-linking provided decoy binding sites for vancomycin and led to a decrease in the susceptibility of *S. aureus* to vancomycin (30). The antibacterial activity of lugdunin targets bacteria by dissipating their membrane potential (31); therefore, changes in the cell wall architecture of VISA and hVISA may also contribute to their higher resistance to lugdunin. Taken together, we demonstrated the epidemiology of lugdunin production among *S. lugdunensis* isolates in Taiwan and revealed its association with genotypes. However, the regulatory mechanism by which lugdunin production is regulated remains unclear.

## SUPPLEMENTAL MATERIAL

Table S1 spectrum.01298-23-s0001.docx Sample sources and antimicrobial susceptibility

ASM does not own the copyrights to Supplemental Material that may be linked to, or accessed through, an article. The authors have granted ASM a non-exclusive, world-wide license to publish the Supplemental Material files. Please contact the corresponding author directly for reuse.
