# Supplementary material for: Lugdunin production and activity in Staphylococcus lugdunensis isolates are associated with its genotypes
Source: Microbiol Spectr. 2023 Sep 21;11(5):e01298-23. doi: 10.1128/spectrum.01298-23 (PMC10580833; doi:10.1128/spectrum.01298-23)
Supplement: Table S1 — Sample sources and antimicrobial susceptibility [file spectrum.01298-23-s0001.docx]

**Supplementary Table S1**

The sample sources and antimicrobial susceptibility results of 202 *S. lugdunensis* isolates

| **Antibiotics** | **No. of non-susceptible isolates (%)** | | |
| --- | --- | --- | --- |
|  | **Total**  **(n = 202)** | **lugdunin-producing**  **(n = 51)** | **Lugdunin non-producing**  **(n = 151)** |
| P | 148 | 37 (25) | 111 (75) |
| OX | 41 | 14 (34.1) | 27 (65.8) |
| CC | 50 | 3 (6) | 47 (94) |
| E | 51 | 3 (5.8) | 48 (94.1) |
| SXT | 4 | 1 (25) | 3 (75) |
| TEC | 0 | 0 (0) | 0 (0) |
| VA | 0 | 0 (0) | 0 (0) |
|  |  |  |  |
| **Sample source** |  |  |  |
| AB | 4 | 0 (0) | 4 (100) |
| AM | 1 | 0 (0) | 1 (100) |
| AS | 6 | 0 (0) | 6 (100) |
| B | 96 | 24 (25) | 72 (75) |
| BF | 3 | 0 (0) | 3 (100) |
| CSF | 1 | 1 (100) | 0 (0) |
| CX | 3 | 1 (33.3) | 2 (66.6) |
| DTS | 25 | 9 (36) | 16 (64) |
| PL | 1 | 0 (0) | 1 (100) |
| PUS | 31 | 10 (32.2) | 21 (67.7) |
| SY | 2 | 0 (0) | 2 (100) |
| TS | 1 | 0 (0) | 1 (100) |
| WD | 18 | 5 (3.6) | 13 (72.2) |
| OTH | 10 | 1 (10) | 9 (90) |

**Abbreviations:** P=Penicillin; OX=Oxacillin; CC=clindamycin; E=erythromycin; SXT=sulfamethoxazole-trimethoprim; TEC=teicoplanin; VA=vancomycin.

AB=Abscess; AM=Amniotic fluid; AS=Ascites; B=Blood; BF=Body fluid; CSF= Cerebrospinal fluid; CX=Endocervix discharge; DTS=Deep tissue; PL=Pleural effusion; PUS=Pus; SY=Synovial fluid; TS=Tissue; WD=Wound; OTH=Other.
